# Supplementary material for: Identification of Amino Acids and Polyphenolic Metabolites in Human Plasma by UHPLC-ESI-QTOF-MS/MS, after the Chronic Intake of a Functional Meal in an Elderly Population
Source: Foods. 2024 Aug 6;13(16):2471. doi: 10.3390/foods13162471 (PMC11354128; doi:10.3390/foods13162471)
Supplement: Supplementary file 1 [file foods-13-02471-s001.zip › foods-3078485-supplementary.pdf]

Table S1. Mass spectral information of amino acids identified in plasma samples.

**Compound**	**R.T. (min)**	**Formula**	**Precursor [M-H]**	**Exact mass**	**Theoretical mass**	**Difference (ppm)**
Glutamic acid	0.422	$C_5 H_9 N O_4$	146.0466	147.0539	147.0532	4.99
Glutamine	1.635	$C_5 H_{10} N_2 O_3$	145.0616	146.0688	146.0691	-2.54
Histidine	0.321	$C_6 H_9 N_3 O_2$	154.0616	155.0688	155.0695	-4.63
Leucine	0.42	$C_6 H_{13} N O_2$	130.0874	131.0943	131.0946	-2.73
Lysine	0.316	$C_6 H_{14} N_2 O_2$	145.098	146.1053	146.1055	-1.85
Phenylalanine	0.523	$C_9 H_{11} N O_2$	164.072	165.0792	165.079	1.48
Proline	0.381	$C_5 H_9 N O_2$	114.0559	115.0632	115.0633	-1.03
Tryptophan	0.894	$C_{11} H_{12} N_2 O_2$	203.0834	204.0905	204.0899	3.21
Tyrosine	0.422	$C_9 H_{11} N O_3$	180.0662	181.0734	181.0739	-2.8
L-Valine	0.388	$C_5 H_{11} N O_2$	116.0715	117.0788	117.079	-1.94

Table S2. Mass spectral information of polyphenolic compounds and metabolites.

Compound	R.T. (min)	Formula	Precursor [M-H]	Exact mass	Theoretical mass	Difference (ppm)
3'-Hydroxymelanettin	6.211	$C_{16} H_{12} O_6$	299.0559	300.063	300.0634	-1.46
3-Hydroxyhippuric acid	3.886	$C_9 H_9 N O_4$	194.0454	195.0533	195.0532	0.78
5-(3',4'-dihydroxyphenyl)-valeric acid	5.815	$C_{11} H_{14} O_4$	209.0813	210.0887	210.0892	-2.61
Citric acid	0.423	$C_6 H_8 O_7$	191.0194	192.0266	192.027	-2.28
4-Ethylphenol	4.324	$C_8 H_{10} O$	121.066	122.073	122.0732	-1.55
Catechol	0.819	$C_6 H_6 O_2$	109.0295	110.0367	110.0368	-0.71
Carnosic acid	6.989	$C_{20} H_{28} O_4$	331.1911	332.198	332.1988	-2.37
Caryatin glucoside	6.381	$C_{23} H_{24} O_{12}$	491.1189	492.1267	492.1268	-0.24
6'-Hydroxyenterolactone	7.260	$C_{18} H_{18} O_5$	313.107	314.1144	314.1154	-3.4
Homovanillic acid	0.891	$C_9 H_{10} O_4$	181.0508	182.0579	182.0579	-0.2
3-Hydroxyphenylvaleric acid	6.784	$C_{11} H_{14} O_3$	193.0868	194.0941	194.0943	-0.77
5-(3'-Methoxy-4'-hydroxyphenyl)-valerolactone	6.419	$C_{12} H_{14} O_4$	221.0816	222.0889	222.0892	-1.42
3,4-Dihydroxytoluene	2.070	$C_7 H_8 O_2$	123.0451	124.0522	124.0524	-1.79
Isopropyl 3-(3,4-Dihydroxyphenyl)-2-hydroxypropanoate	6.388	$C_{12} H_{16} O_5$	239.0925	240.0996	240.0998	-0.65
Hippuric acid	1.632	$C_9 H_9 N O_3$	178.0508	179.0581	179.0582	-0.66
Protocatechuic acid	1.868	$C_7 H_6 O_4$	153.0192	154.0265	154.0266	-0.52
Dicaffeoyl quinic acid	6.717	$C_{25} H_{24} O_{12}$	515.1195	516.1272	516.1268	0.86
Phenacetylglycine	3.245	$C_{10} H_{11} N O_3$	192.0664	193.0736	193.0739	-1.43
Equol	6.992	$C_{15} H_{14} O_3$	241.0874	242.0941	242.0943	-0.82
2-Hydroxyphenylacetic acid	4.126	$C_8 H_8 O_3$	151.0401	152.0473	152.0473	-0.33
Vanillin	2.744	$C_8 H_8 O_3$	151.0402	152.0475	152.0473	1.13
Carnosol	6.720	$C_{20} H_{26} O_4$	329.175	330.1823	330.1831	-2.4
Azaleatin	0.320	$C_{16} H_{12} O_7$	315.0508	316.0588	316.0583	1.49

4'-O-Methylequol	6.485	$C_{16} H_{16} O_3$	255.1025	256.1098	256.1099	-0.36
4'-O-Methylcatechin	3.957	$C_{16} H_{16} O_6$	303.0874	304.0947	304.0947	0.16
Benzoic acid	5.138	$C_7 H_6 O_2$	121.0295	122.0367	122.0368	-0.77
